# Skin α-synuclein deposits differ in clinical variants of synucleinopathy: an in vivo study

**DOI:** 10.1038/s41598-018-32588-8

**Published:** 2018-09-24

**Authors:** V. Donadio, A. Incensi, O. El-Agnaf, G. Rizzo, N. Vaikath, F. Del Sorbo, C. Scaglione, S. Capellari, A. Elia, M. Stanzani Maserati, R. Pantieri, R. Liguori

**Affiliations:** 1 IRCCS Istituto delle Scienze Neurologiche, Bologna, Italy; 2 Life Sciences Division, College of Science and Engineering, Hamad Bin Khalifa University (HBKU), Education City, Qatar Foundation, Doha, Qatar; 3 Dipartimento di Scienze Biomediche e Neuromotorie, Università di Bologna, Bologna, Italy; 4 Fondazione IRCCS Istituto Neurologico Carlo Besta, Milano, Italy

## Abstract

We aimed to characterize *in vivo* α-synuclein (α-syn) aggregates in skin nerves to ascertain: 1) the optimal marker to identify them; 2) possible differences between synucleinopathies that may justify the clinical variability. We studied multiple skin nerve α-syn deposits in 44 patients with synucleinopathy: 15 idiopathic Parkinson’s disease (IPD), 12 dementia with Lewy Bodies (DLB), 5 pure autonomic failure (PAF) and 12 multiple system atrophy (MSA). Ten healthy subjects were used as controls. Antibodies against native α-syn, C-terminal α-syn epitopes such as phosphorylation at serine 129 (p-syn) and to conformation-specific for α-syn mature amyloid fibrils (syn-F1) were used. We found that p-syn showed the highest sensitivity and specificity in disclosing skin α-syn deposits. In MSA abnormal deposits were only found in somatic fibers mainly at distal sites differently from PAF, IPD and DLB displaying α-syn deposits in autonomic fibers mainly at proximal sites. PAF and DLB showed the highest p-syn load with a widespread involvement of autonomic skin nerve fibers. In conclusion: 1) p-syn in skin nerves was the optimal marker for the *in vivo* diagnosis of synucleinopathies; 2) the localization and load differences of aggregates may help to identify specific diagnostic traits and support a different pathogenesis among synucleinopathies.

## Introduction

A common feature of synucleinopathies is the pathological accumulation of misfolded α-synuclein (α-syn) leading to neuron dysfunction and death^1^. Based on brain post-mortem studies different α-syn strains possibly expressing specific molecular conformations have been proposed mainly in idiopathic Parkinson’s disease (IPD)^2,3^. In addition, a recent study demonstrated that α-syn strains extracted from the brain of Multiple System Atrophy (MSA) patients showed different prion properties than the strains extracted from the brain of IPD patients^4^. These findings may suggest that distinct deposits of pathological α-syn are involved in neurodegenerative diseases possibly providing the heterogeneity of synucleinopathies^2,5^ as described in prion disorders^6^. However, the pathogenetic mechanism underlying synucleinopathies is far from being fully understood because of the unavailability of a systematic study of α-syn aggregations in different clinical phenotypes and the lack of *in vivo* data allowing to analyse abnormal α-syn aggregates before the widespread diffusion and the late maturation of these deposits^7^.

Skin biopsy is a promising diagnostic tool for the *in vivo* diagnosis of synucleinopathies^8–14^ but a study simultaneously testing different α-syn epitopes to detect abnormal deposits in all clinical variants of synucleinopathy is lacking. Hypothesizing the involvement of different α-syn deposits raises the possibility that a single marker could be unsuitable for disclosing abnormal deposits in all clinical variants. Thus a systematic study of α-syn deposits distribution in clinical variants of synucleinopathy is also needed for diagnostic purposes and to support skin biopsy as a promising diagnostic tool for these disorders.

This study aimed to characterize abnormal α-syn deposits in skin nerves by immunofluorescence to ascertain the *in vivo* existence of different aggregates in variants of synucleinopathy. It may therefore contribute to clarifying in synucleinopathies: 1) the optimal diagnostic marker to disclose skin nerves α-syn deposits in different variants; 2) whether an *in vivo* different distribution of α-syn deposits may justify the clinical variability.

## Materials and Methods

We studied 44 patients with synucleinopathy including 15 IPD patients fulfilling established diagnostic criteria^15^, 12 patients who met the clinical diagnostic criteria for probable dementia with Lewy bodies (DLB-5 of them presenting with orthostatic hypotension)^16^, 5 fulfilling diagnostic criteria for pure autonomic failure (PAF)^17^ and 12 for MSA (5 MSA-P and 7 MSA-C)^18^ (Table 1 reports demographic data and the clinical profiles of the patients included in the study). Disease duration of recruited patients did not differ among different variants (p > 0.1). Recruited patients were well characterized since the clinical diagnosis was supported by abnormal laboratory tests showing cardiac postganglionic sympathetic denervation (123-I-MIBG)^19^, dopaminergic nigrostriatal abnormalities (123I-ioflupane-DatScan)^20^ or brainstem and cerebellum atrophy and/or the hot-cross bun sign (brain MR)^21,22^. Ten age-matched healthy subjects served as controls. The procedures used were approved by the local Human Ethics Committee (Comito Etico Indipendente-AUSL Bologna, cod. 13004) and followed the Helsinki Declaration regarding international clinical research involving human beings. All participants gave their written informed consent to be included in the study.

**Table 1:** Clinical and laboratory findings of patients.

No.	IPD	DLB	PAF	MSA	Controls
	15	12	5	12	10
**Age**					
Mean ± SD years	70 ± 3	75 ± 6	67 ± 10	66 ± 9	70 ± 3
**Sex**					
male:female	08:07	08:04	04:01	08:03	06:04
**Dis. Dur**.					
Mean ± SD years	6 ± 4	4 ± 2	7 ± 1	5 ± 1	—
OH (%)	0	42	100	100	0
UPDRS	28 ± 8	11 ± 3	0	25 ± 3 (5°)	0
RBD (%)	15	80	0	82	0
Abnormal Cardiac MIBG (%)	100 (3)	100 (4)	100	0 (4)	—
Abnormal DatScan (%)	100	100 (10)	0	60 (7)	—
Brainstem abnormalities (MR) (%)	0	0	0	100	—

Dis.Dur. = disease duration; UPDRS-III = motor examination; OH = orthostatic hypotension; RBD = rem behavioral sleep disorder; the number in brackets represents the number of patients in whom the test was performed; °patients with MSA-P variant.

### Skin biopsy

Following a previously described protocol^11,23^ 3 mm punch biopsies were taken from proximal and distal hairy skin sites. The proximal site included the cervical C7 paravertebral area whereas distal sites were located in the thigh (15 cm above the patella) and distal leg (10 cm above the lateral malleolus). Two samples were taken in each skin site 3–4 centimetres away^11,23^. According to previously published procedures^15,24^, skin samples were immediately fixed in cold Zamboni’s fixative and kept at 4 °C overnight. Skin sections were obtained using a freezing sliding microtome (HM550, Thermo Scientific, Walthan, MA, USA).

#### Immunofluorescence characterization of skin nerve α-syn aggregates

Ten μm skin sections were double-immunostained overnight (unless differently specified) with a panel of primary antibodies against α-syn epitopes and the rabbit or mouse pan-neuronal marker protein gene product 9.5 (PGP). The correspondence between α-syn markers and PGP helped to verify the intraneuronal α-syn staining excluding non-specific dot-like staining often experienced in patients and controls inside membranes, sweat glands tubules or vessel endothelium^11^. A rule to identify abnormal α-syn aggregates was the co-localization of PGP and antibodies against abnormal α-syn epitopes expression of C-terminal post-translational modifications or amyloid fibrils. Furthermore, different primary antibodies against normal or abnormal α-syn and ubiquitin were also double stained to characterize abnormal α-syn deposits. A triple combination of antibodies was not allowed because of only two different species of antibodies available (i.e. rabbit or mouse). Primary antibodies used in this study (reported in Table 1-supplemental file) included antibodies against the native form of α-syn (n-syn) or α-syn core (NAC) and against C-terminal α-syn epitopes involved in post-translational modifications such as rabbit or mouse (immunostained for only 1 hour) phosphorylation α-syn at serine 129 (p-syn) and tyrosine 125 (pY-syn), nitration at tyr125–133 (nY-syn). Amyloid α-syn fibrils were characterized by using a non-commercial antibody (syn-F1)^25^, whereas advanced glycation end products (AGEs) residues that may be linked to abnormal α-syn deposits^26^ were disclosed by a specific marker. Furthermore, a specific mouse monoclonal antibody against full-length ubiquitin a.a. 1–76 (m-ub, 1:100, Santa Cruz, USA; cod. Sc-8017) was used to detect ubiquitin deposits often associated with α-syn fibrils^27^. We have tried an overnight incubation of primary antibodies^25^ but the final staining on skin sections was poor. In addition the final dilution of primary antibodies was established after testing a large range of dilutions. A non-commercial antibody to detect oligomeric forms of α-syn (syn-O)^25^ was also tested but it was not systematically used in this study because preliminary experiments showed a frequent co-localization with NAC in skin nerves of controls and patients in all dilution used (1: 5000 and 1:10.000, data not shown).

Sections were then washed and secondary antibodies were added for an incubation of one hour. As secondary antibodies, an anti-mouse or rabbit Alexa Fluor(R) 488 and anti-rabbit or mouse Jackson cyanine dye fluorophores 3.18 (1:200 or 1:400; Jackson ImmunoResearch, West Grove, PA, USA; cod. 715–545–150 and 711-545-152 for mouse and rabbit AlexaFluor488 respectively and 715-165-150 and 711-165-152 for mouse and rabbit cyanine 3) were used.

#### Co-localization study

**Figure 1:**
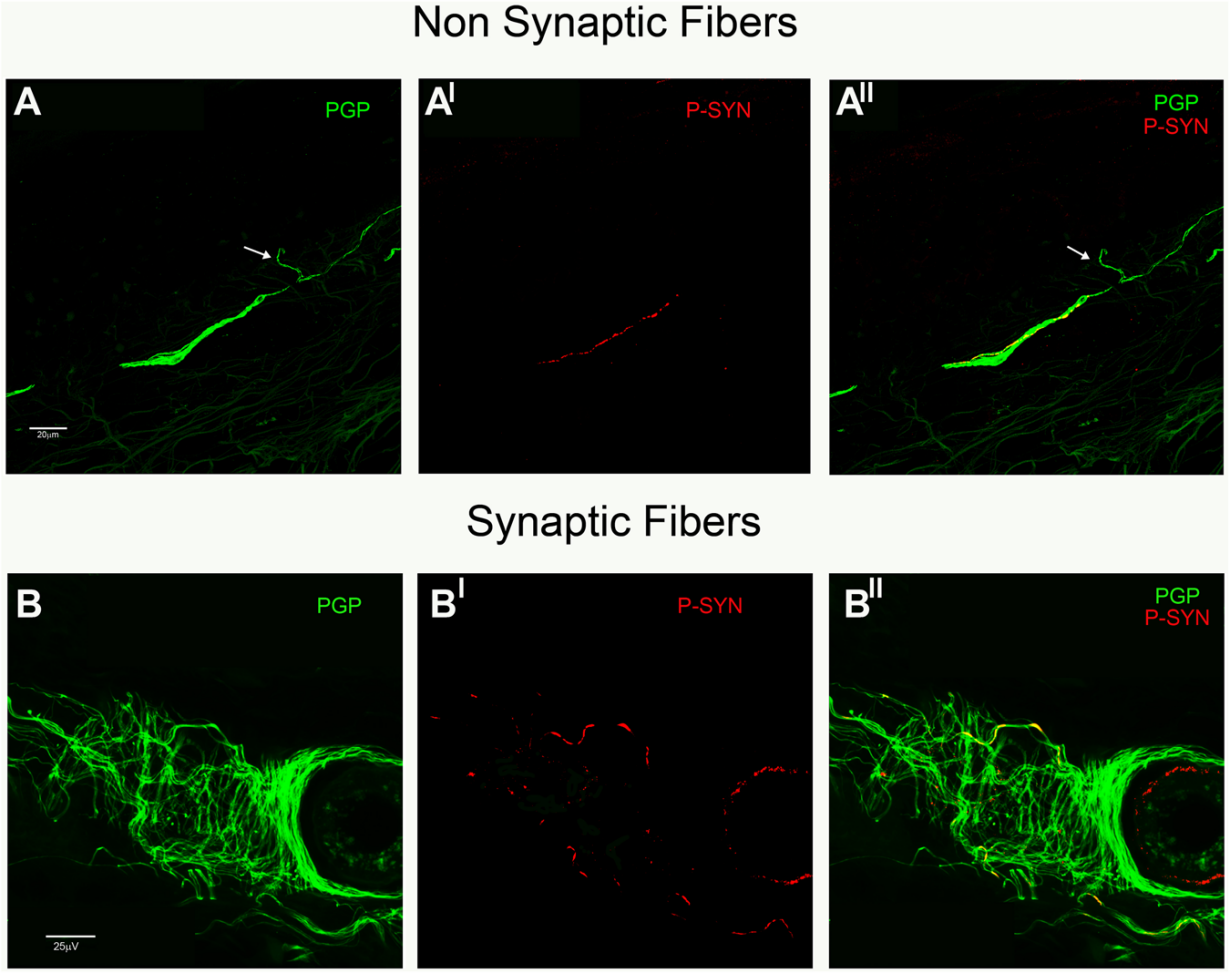
Abnormal intraneural p-syn aggregates in non-synaptic and synaptic fibers. Examples of phosphorylated α-synuclein aggregates in a non-synaptic fiber of a patient with MSA (**A**) and synaptic fibers of a DLB patient (**B**) disclosed by confocal microscope (x400). (**A**)Subepidermal plexus close to the epidermis as confirmed by an isolated epidermal free-ending PGP-ir fibers (arrow) was identified by a PGP staining (**A**). The plexus showed a positive phosphorylated α-syn (A^I^) as neuritic inclusion demonstrated by the merged image (A^II^). (**B**)Nerve fibers innervating a sweat gland tubule were depicted by PGP (**B**). Some of these fibers showed aggregates of p-syn (B^I^) as intraneural inclusions in the merged image (B^II^).

Digital images were acquired using a laser-scanning confocal microscope and subsequently projected to obtain a 3D digital image by a computerized system (Nikon confocal microscopy, Eclipse Ti A1). The sections selected for the analysis include frames of 0.25 μm on a Z-stack plan at the appropriate wavelengths for secondary antibodies with a x400 or x600 plan apochromat objective. The co-localization between two different fluorescent signals was first judged absent or present on a single 0.25 μm frame by the agreement of two authors with major expertise in immunoflorescence analysis (DV and IA). As the co-localization was considered present it was calculated by NIS-elements Sofware (Nikon, Tokio, Japan) to obtain the Pearson’s coefficient (Rp) from −1 = the two signals changed in the opposite direction (absent co-localization) to 1 = the two signals changed in the same direction (perfect co-localization); a value of >0 was taken as significant co-localization^28^. The analysis was made for synaptic and non-synaptic fibers. Synaptic fibers were in proximity of terminal nerve endings directed to skin vessels (SV), sweat glands (SG) or in the muscle arrector pilorum (MAP), whereas non-synaptic fibers included axons of nerve plexuses distant from the innervation target (Fig. 1). Abnormal α-syn aggregates were characterized by using several antibodies in consecutive skin sections expressing the same aggregate because, as previously specified, we were unable to combine more than 2 different antibodies against α-syn in the same skin section.

#### Spatial characterization of α-syn aggregates

The spatial distribution of abnormal aggregates was also analysed considering p-syn staining that showed the highest specificity in identifying these aggregates. The following parameters were considered:

#### P-syn sample rate

It was expressed by dividing the total number of skin fibers positive for p-syn by the number of skin samples analysed in each patient. P-syn sample rate was calculated for synaptic and non-synaptic fibers.

#### P-syn occurrence in consecutive skin sections

The p-syn immunoreactivity in skin nerves in a broad skin area was analysed considering 6 consecutive free-floating thick skin sections of 50 μm of the same skin sample (300 μm). The percentage of skin sections showing p-syn positivity was reported: 100% expressed a p-syn positivity throughout all 6 skin sections.

#### Proximal/distal p-syn gradient

The p-syn positivity for each skin site (considering both skin samples) was calculated in all patients with the same clinical variant and expressed as percentage: 100% represents the positivity in all patients.

### Statistical analysis

Statistical analyses were performed using SPSS 24.0 for Windows. For the analysis of continuous variables we used Kolmogorov–Smirnov test to verify the normal distribution of the data. One-way analysis of variance (ANOVA) followed by a post hoc Bonferroni test was performed for comparison of normally distributed data. The Kruskall–Wallis test was used to test whether significant intergroup differences occurred, when the variables were not normally distributed or the sample size was too small. Where significant differences were found, pair-wise comparisons were performed using a post hoc Mann–Whitney U-test, and resulting P values were corrected for multiple comparisons according to the Bonferroni method. We used χ^2^ test for the analysis of categorical variables. For all analyses, significance was assumed as corrected P < 0.05.

## Results

### Skin nerve α-syn deposits

#### Controls

**Figure 2:**
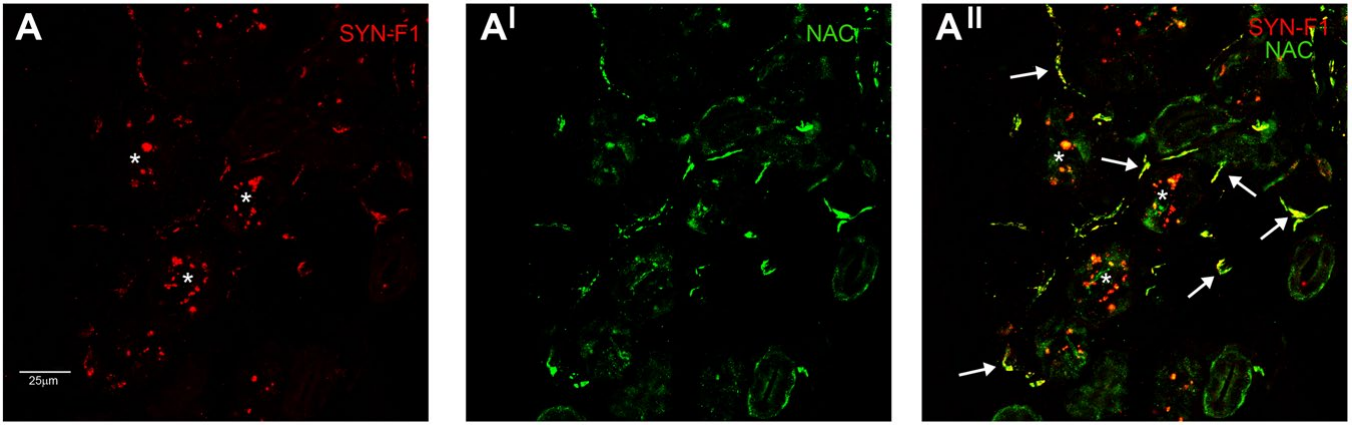
NAC and syn-F1 co-localization in a healthy control. Confocal microscope images (X 200) showing a weak syn-F1 staining in sweat gland of healthy control. The syn-F1 staining co-localized with NAC (arrows in A^II^) demonstrating that this signal is likely a non-specific signal arising from native α-syn. The asterisks represents sweat tubules autofluorescence.

NAC and n-syn were homogenously expressed in the dermal annexes’ innervation (MAP, SG, SV and hair follicles), whereas no staining was found in the epidermal fibers. Skin plexuses were usually devoid of native α-syn staining although occasionally a signal was found. NAC and syn-n showed essentially the same result. A weak co-staining was occasionally found between NAC and syn-F1 in dermal annexes fibers (Fig. 2) whereas p-syn, AGEs, nY-syn and pY-syn staining were not found in controls.

#### Patients with synucleinopathy

**Figure 3:**
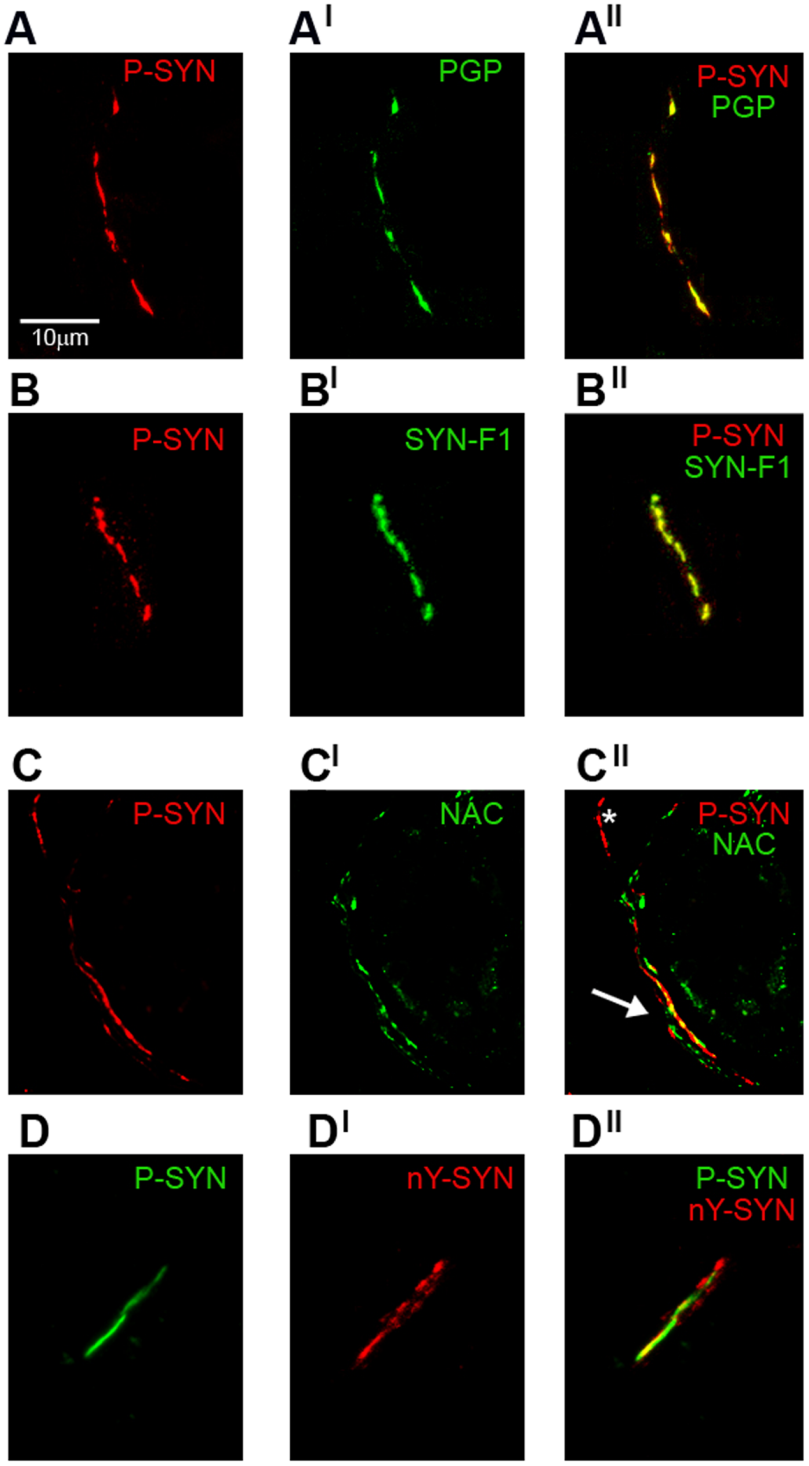
Abnormal α-syn aggregates characterized by a combination of different primary antibodies. Confocal microscope study (x400) of α-syn aggregates in patients with different variants of synucleinopathy based on a co-localization between p-syn and a neuronal marker (i.e. PGP) or antibodies against abnormal α-syn epitopes expression of C-terminal post-translational modifications or amyloid fibrils (syn-F1). (**A**) P-syn demonstrated an excellent co-localization with PGP (A^I^) in a nerve plexus supporting the intraneural deposition of abnormal α-syn aggregates (A^II^). (**B**) The co-localization in a nerve plexus between p-syn and syn-F1 (B^I^) found in the majority of analysed deposits supporting the fibrillar nature of these aggregates (B^II^). (**C**) Sudomotor fibers around a sweat tubule marked by NAC (C^I^) were co-localized with p-syn (arrow in C^II^) although other sudomotor fibers stained by p-syn were devoid of NAC staining (asterisk in C^II^). (**D**) NY-syn staining was occasionally seen in non-synaptic fibers (D^I^) and this staining co-localized with p-syn (D^II^). The four different coexistent fibrillar and non-fibrillar α-syn deposits found in skin nerves were similarly distributed among different clinical phenotypes. Nevertheless, these deposits showed important differences in specific variants of synucleinopathy such as their localisation (i.e. only in somatosensory skin fibers in MSA - see Table 2) or the widespread involvement of autonomic annexes (i.e. in PAF and DLB - see Fig. 4). These differences may support a different pathogenesis among synucleinopathies helping to identify specific diagnostic traits.

**Figure 4:**
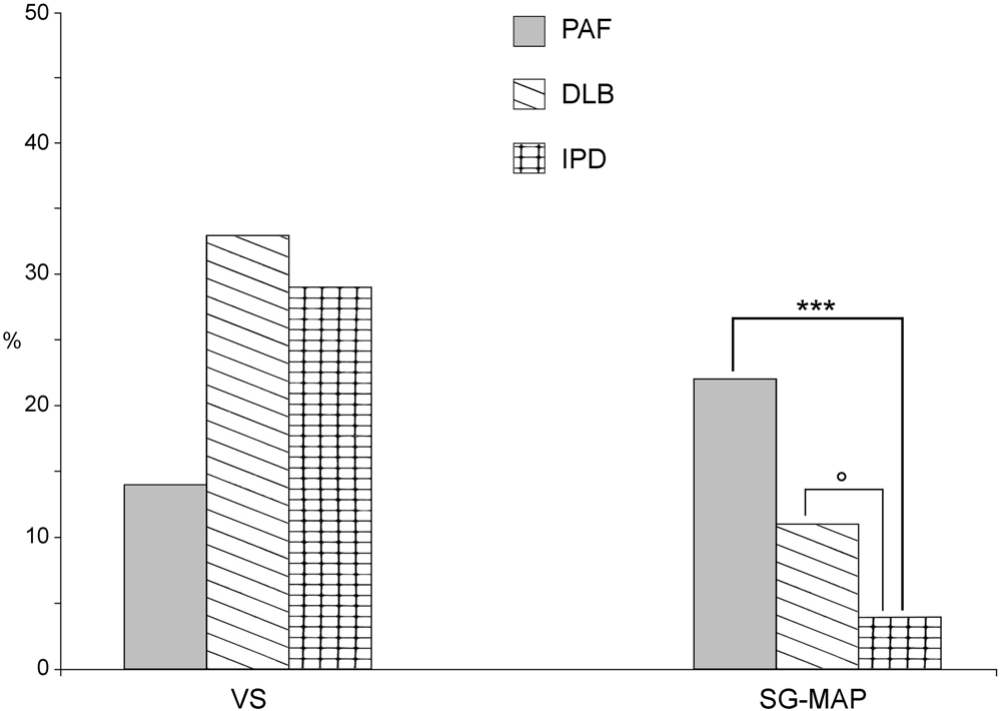
Distribution of intraneural abnormal α-syn deposits in autonomic annexes. The pattern of p-syn distribution among autonomic annexes disclosed a non-significant difference of deposits around skin vessels (SV) in IPD, PAF and DLB. By contrast abnormal α-syn deposits were significantly higher in sweat glands (SG) and muscle arrector pilorum (MAP) of PAF than in IPD whereas DLB showed an intermediate degree of involvement. These results underlined a widespread extension of deposits in autonomic annexes of patients with autonomic symptoms such as PAF and DLB. ***p < 0.001; °p = 0.07.

Abnormal α-syn deposits were found in all patients except 4 with MSA-C. A total of 185 skin nerve α-syn deposits were identified: 50 in IPD, 65 in DLB, 40 in PAF and 30 in MSA (Table 2; Fig. 1). P-syn staining showed the highest rate of positivity and specificity since it was never found in controls. Accordingly, p-syn staining was selected as the main marker to characterize abnormal skin α-syn aggregates in co-localization studies. P-syn deposits were often co-localized with syn-F1 (around 90% of deposits) and less with NAC (around 30% of deposits) in all variants of synucleinopathy (Table 2). Occasionally, syn-F1 showed a weak staining in autonomic nerve endings without p-syn but this represents a non-specific signal arising from native α-syn since it was co-localized with NAC and found also in controls (Fig. 2). nY-syn staining was occasionally seen in non-synaptic fibers without differences among synucleinopaties (1 fiber stained in each disorder), whereas AGEs, pY-syn and ubiquitin did not stain any α-syn deposits. Co-localization studies showed four coexisting α-syn aggregates in consecutive sections (Fig. 3): a) non-fibrillar aggregates (i.e. stained only by p-syn; Fig. 3A); b) fibrillar aggregates (i.e. positive only for syn-F1 and p-syn; Fig. 3B); c) fibrillar aggregates showing native epitopes (i.e. positive also for NAC; Fig. 3C); d) fibrillar aggregates positive for nitrate α-syn but not for native staining (Fig. 3D). MSA showed abnormal aggregates only in somatosensory (i.e. non synaptic) fibers mainly of the subepidermal plexus (Fig. 1A) with usually a dot-like staining. This pattern differed from the remaining synucleinopathies (IPD, DLB and PAF) by showing p-syn deposits in autonomic fibers and plexuses close to autonomic annexes (Table 2; Fig. 1B) with a more homogenous staining, although autonomic fibers were differently affected in IPD showing p-syn deposits mainly around SV and PAF presenting with a widespread extension of deposits also involving GH and MAP. Abnormal aggregates in autonomic annexes showed an intermediate degree of extension in DLB (Fig. 4).

**Table 2:** P-syn co-localization analysis in synaptic and non synaptic fibers.

*IPD*	No. deposits		syn-F1 co-localization		NAC co-localization	
			%	P.C.	%	P.C.
synaptic fibers	29		88	0.7 ± 0.2	42	0.3 ± 0.2
non synaptic fibers	21		92	0.8 ± 0.1	10	0.5 ± 0.1
Tot	**50**	**Mean ± SD**	**90**	**0.8 ± 0.1**	**29**	**0.4 ± 0.2**
***DLB***						
synaptic fibers	54		82	0.8 ± 0.1	33	0.3 ± 0.2
non synaptic fibers	11		100	0.8 ± 0.1	5	0.2 ± 0.1
Tot	**65**	**Mean ± SD**	**86**	**0.8 ± 0.1**	**32**	**0.3 ± 0.1**
***PAF***						
synaptic fibers	22		100	0.8 ± 0.1	39	0.3 ± 0.1
non synaptic fibers	18		82	0.8 ± 0.1	14	0.3 ± 0.1
Tot	**40**	**Mean ± SD**	**90**	**0.8 ± 0.1**	**30**	**0.3 ± 0.1**
***MSA***						
synaptic fibers	0		0	0	0	0
non synaptic fibers	30		90	0.9 ± 0.1	18	0.3 ± 0.01
Tot	**30**	**Mean ± SD**	**90**	**0.9 ± 0.1**	**18**	**0.3 ± 0.01**

Values did not show a significant difference; P.C. = Pearson coefficient.

### Spatial distribution of α-syn deposits

#### P-syn sample rate

The amount of p-syn fibers per skin sample was higher in PAF and DLB than IPD and MSA (Table 3). Furthermore, MSA was characterized by absent p-syn deposits in autonomic synaptic fibers.

**Table 3:** Spatial distribution of p-syn skin fibers

*IPD*	P-syn sample rate	Serial p-syn occurrance
	fiber/sample	%
synaptic fibers	0.4 ± 0.3	40
non synaptic fibers	0.6 ± 0.5	45
Mean ± SD	0.4 ± 0.3*^	**85**
***DLB***		
synaptic fibers	0.5 ± 0.4	30
non synaptic fibers	1.5 ± 0.9	70
Mean ± SD	**0.7 ± 0.4**	**100**
***PAF***		
synaptic fibers	0.3 ± 0.1	40
non synaptic fibers	4.1 ± 1.9	60
Mean ± SD	**1.2 ± 0.6**	**100**
*MSA*		
synaptic fibers	0	0
non synaptic fibers	0.5 ± 0.3	83
Mean ± SD	**0.5 ± 0.3**	**83**

*p < 0.05 IPD vs DLB; ^p < 0.05 IPD vs PAF.

#### P-syn occurrence in consecutive skin sections

**Figure 5:**
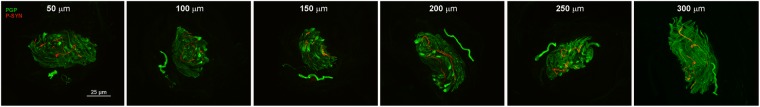
P-syn occurrence in consecutive skin sections of a PAF patient. Immunoreactivity of α-synuclein phosphorylated at serine 129 (p-syn) in a single nerve fiber in a broad skin area was analysed considering 6 consecutive free-floating thick skin sections of 50 μm of the same skin sample. A patient with PAF showed a persistent occurrence of p-syn staining in all consecutive skin sections supporting a regular distribution along skin nerves.

Six different skin samples were analysed in PAF, 8 in DLB and MSA and 9 IPD. DLB and PAF showed the persistent occurrence of p-syn staining along skin nerves (Fig. 5), whereas in IPD and MSA p-syn deposits showed a lower occurrence (Table 3) suggesting a more irregular distribution along skin nerves.

#### Proximal/distal p-syn gradient

**Figure 6:**
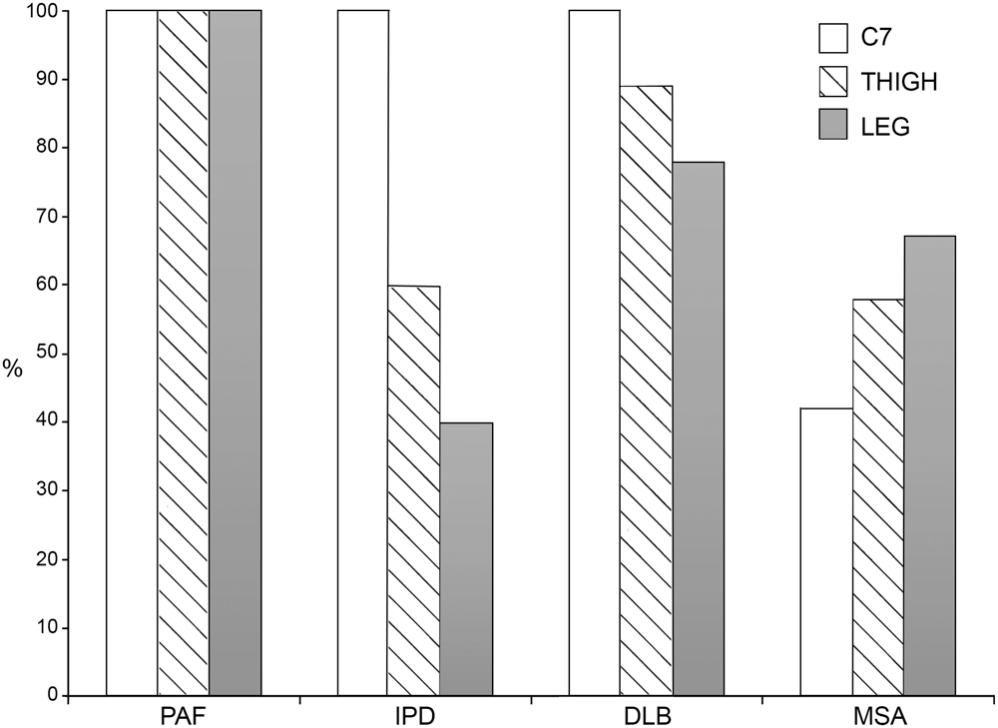
Proximal/distal p-syn gradient. The figure illustrates the different pattern of p-syn distribution throughout proximal and distal skin sites in clinical variants of synucleinopathy. (**A**) PAF showed a homogenous p-syn positivity in proximal and distal sites. (**B**) IPD (corrected p < 0.01) and DLB (p = 0.05; corrected p = 0.1) displayed a p-syn proximal-distal gradient with higher positivity in proximal sites, mainly the cervical area. (**C**) MSA showed an opposite pattern of skin nerve p-syn with higher positivity in distal skin sites although the difference was not significant (p > 0.4).

Three different patterns of p-syn distribution were found: (1) the homogenous distribution of p-syn positivity in proximal and distal skin sites in PAF (Fig. 6A); (2) the higher positivity of p-syn in proximal sites, mainly C7 in IPD (corrected p < 0.01) and DLB (p = 0.05; corrected p = 0.1) (Figs 3 and 6B) the higher p-syn positivity in distal skin sites, mainly the leg in MSA although the difference was not significant (p > 0.4) (Fig. 6C).

## Discussion

The main results of our study were: 1) p-syn as the most sensitive and specific marker of abnormal α-syn deposits in skin nerves for the *in vivo* diagnosis of synucleinopathies; 2) different coexistent fibrillar and non-fibrillar α-syn deposits were found in clinical variants of synucleinopathy; 3) MSA displayed a peculiar pattern of abnormal deposits only found in somatosensory skin fibers and PAF-DLB showed the highest load of deposits with a widespread involvement of autonomic annexes. These differences may help to identify specific diagnostic traits and may support a different pathogenesis among synucleinopathies.

### Abnormal deposits of α-syn in skin nerves were optimally disclosed by the antibody against α-syn phosphorylation at serine 129

Skin biopsy by means of an immunofluorescence technique is a promising tool for the *in vivo* diagnosis of synucleinopathies since this technique is straightforward, inexpensive and minimally invasive with minor discomfort for the patient. However, a systematic study to test the sensitivity and specificity of different antibodies in disclosing misfolded, abnormal α-syn deposits, as reported in the brain of post-mortem studies^2,29,30^, is lacking *in vivo*. Our data demonstrated that the antibody against phosphorylation at serine 129 showed the optimal sensitivity and specificity in disclosing Lewy neuritis in skin nerves in different variants of synucleinopathy as previously reported by independent groups in single clinical entities^9–14,23^. The sensitivity in disclosing α-syn deposits was lower in MSA mainly because of MSA-C showing no skin deposits in the majority of analysed patients. However, a more focused study involving a larger cohort of patients is needed to confirm the difference in disclosing skin p-syn deposits between MSA-C and MSA-P. The antibody against α-syn fibrils (syn-F1) presented with a comparable sensitivity in disclosing the abnormal synuclein deposits but it was less specific than p-syn since it was also found in controls. The other antibodies against α-syn that we have tested did not show appreciable sensitivity in disclosing abnormal deposits in the skin nerves differently from post-mortem brain studies and their use is not recommended for the *in vivo* diagnosis of synucleinopathies by skin biopsy. These data were supported by previous works showing that α-syn phosphorylation at ser129 was a diffuse pathological event in synucleinopathies^30^, whereas α-syn nitration and glycation were found mainly associated with brain Lewy body^31^. Skin α-syn deposits did not show a positive staining for ubiquitin suggesting that ubiquination which may promote the degradation of deposits by targeting them for proteasome^32,33^, could occur in different compartments of the neuron such as the cell body where the ubiquitin-proteasome system mainly works. However, since we are unable to stain brain tissue we cannot exclude that a different staining of antibodies against AGEs, pY-Syn, nY-Syn and ubiquitin between brain and skin tissues could be due to a technical difference even if in the skin sections we have tested several immunofluorescence protocols reported to work in the brain sections.

### Different coexistent fibrillar and non-fibrillar α-syn deposits were found in clinical variants of synucleinopathy

Our data demonstrated that synucleinopathies showed four different coexistent aggregates in skin nerves similarly distributed among different clinical phenotypes. Since investigated patients did not show different disease duration our data may support the conclusion that disease duration is correlated to the type of α-syn aggregates probably representing different stages of maturity of Lewy pathology^23,34,35^. However, future studies investigating patients with different disease duration are needed to confirm this conclusion since several lines of research are in disagreement with it: 1) distinct brain α-syn strains with different affinities to neurons, glial cells or astrocytes targeting specific cerebral circuits in human brain have been described^29,36^; 2) the injection in the mouse brain of structurally different α-syn strains (oligomers, ribbons and fibrils) demonstrated differential seeding propensity leading to distinct histopathological and behavioural phenotypes^37^; 3) α-syn strains extracted from the brain of MSA and IPD patients demonstrated different seeding properties^4,38^.

Nevertheless, important differences were achieved in different clinical variants related to the p-syn load and the cell-type specific distribution of p-syn aggregates: somatosensory fibers in MSA but autonomic fibers in the other variants of synucleinopathy. Interestingly these variants of synucleinopathy (i.e. PAF, DLB and IPD) showed a different load and widespread involvement of autonomic fibers in relationship to the presence of autonomic symptoms (i.e. orthostatic hypotension-OH). The widespread involvement of autonomic annexes, i.e. SV, GH and MAP, characterized patients with OH such as PAF whereas p-syn deposits were lower and mainly localized around SV in patients without OH (i.e. IPD). DLB displayed an intermediate involvement of autonomic annexes and OH was found in approximately half of those patients.

### MSA showed a peculiar pattern of skin misfolded α-syn aggregates

MSA showed a selective involvement of somatosensory fibers as already recently reported^14^ and presented a peculiar pattern of abnormal aggregates in comparison to other synucleinopathies since OH was not associated with the involvement of autonomic fibers. These findings may support a selective cell/neuronal vulnerability in synucleinopathies possibly related to the genetic profile of the patients (i.e. host) predisposing the deposition of misfolded aggregates of α-syn in specific cells^39,40^ as described in prion disorders^41^. This conclusion was suggested by considering that similar types of abnormal aggregates were found in different skin cells and patients with different clinical variants. The specific p-syn deposits in skin somatosensory fibers may be a useful biomarker helping to differentiate MSA from other synucleinopathies even in the early stages of the disease when an isolated sleep symptom is present, i.e. REM sleep behaviour disorder (RBD). In fact, the majority of patients with RBD without motor dysfunctions showed abnormal p-syn deposits in skin nerves^42,43^.

### This work had the following limitations

(1) abnormal α-syn deposits were characterized in consecutive thin (10 μm) skin sections. The assumption being that the same deposits may not change immunofluorescence staining in consecutive thin sections. Similar results achieved in all clinical variants underlined that our data are reproducible and the assumption is accurate; (2) no antibodies against N-terminus α-syn epitopes were used. A recent work showed that N-terminus antibodies efficiently differentiated misfolded α-syn deposits in MSA from other clinical variants supporting a different conformation of misfolded deposits^44^. A future *in vivo* study targeting N-terminal epitopes is needed to ascertain this important point; (3) the number of PAF patients recruited for this study is limited but the number of aggregates studied is similar to the other variants of synucleinopathy; 4) although we studied a high number of abnormal intraneural α-syn deposits investigated subjects were fewer. For this reason this study should be considered as a pilot study and our main results need to be confirmed in a larger cohort of patients.

## Electronic supplementary material

Dataset 1
