# Supplementary material for: Skin α-synuclein deposits differ in clinical variants of synucleinopathy: an in vivo study
Source: Sci Rep. 2018 Sep 24;8:14246. doi: 10.1038/s41598-018-32588-8 (PMC6155202; doi:10.1038/s41598-018-32588-8)
Supplement: Supplementary file 1 — Dataset 1 [file 41598_2018_32588_MOESM1_ESM.docx]

**Skin α-synuclein deposits differ in clinical variants of synucleinopathy: an in vivo study**

Donadio V*, MD^1^, Incensi A, BSc^1^, El-Agnaf O, MD^2^, Rizzo G, MD^1,3^, Vaikath N, MD^2^, Del Sorbo F, MD^4^, Scaglione C, MD^1^, Capellari S, MD^1,3^, Elia A, MD^4^, Stanzani Maserati M, MD^1^, Pantieri R, MD^1^, Liguori R, MD^1,3^

1. IRCCS Istituto delle Scienze Neurologiche, Bologna, Italia
2. Life Sciences Division, College of Science and Engineering, Hamad Bin Khalifa University (HBKU), Education City, Qatar Foundation, Doha, Qatar
3. Dipartimento di Scienze Biomediche e Neuromotorie, Università di Bologna, Italia
4. Fondazione IRCCS Istituto Neurologico Carlo Besta, Milano, Italia

**Address for correspondence:** Dr Vincenzo Donadio – IRCCS Istituto delle Scienze Neurologiche, Bologna, Italia, UOC Clinica Neurologica, via Altura 3, 40139 Bologna, (Italy) – Tel: ++39/051/4966113 - e-mail: [vincenzo.donadio@unibo.it](mailto:vincenzo.donadio@unibo.it)

| **Table 1 suppl. List of primary antibodies against α-synuclein used in this study** | | | | |  | |  | |
| --- | --- | --- | --- | --- | --- | --- | --- | --- |
|  |  |  |  |  | |  | |  |
| **Antibody** | **Host Species** | **Epitope** | **α-synuclein Residues** | **Dilution** | | **Source** | |  |
|  |  |  |  |  | |  | |  |
| **ab51253** | Rabbit monoclonal | phosporylated serine (p-syn) | 129 | 1:500 | | Abcam | |  |
|  |  |  |  |  | |  | |  |
| **825701** | Mouse monoclonal | phosporylated serine (p-syn) | 129 | 1:4000 | | Biolegend | |  |
|  |  |  |  |  | |  | |  |
| **TIP-SN-P08** | Rabbit polyclonal | non-amyloid-b component (NAC) | 75-91 | 1:1000 | | Cosmo Bio Co. | |  |
|  |  |  |  |  | |  | |  |
| **AB5038** | Rabbit polyclonal | Native (n-syn) | 111-131 | 1:1000 | | Millipore | |  |
|  |  |  |  |  | |  | |  |
| **NBP1-26380** | Mouse monoclonal | nitrated-alpha synuclein (nY-syn) | 125 and 133 | 1:1000 | | Novus Biological | |  |
|  |  |  |  |  | |  | |  |
| **Ab10789** | Rabbit polyclonal | phosporylated tyrosine (pY-syn) | 125 | 1:500 | | Abcam | |  |
|  |  |  |  |  | |  | |  |
|  | Mouse monoclonal | amyloid α-synuclein fibrils (syn-F1) |  | 1:500 | | non-commercial antibody | |  |
|  |  |  |  |  | |  | |  |
| **KAL-KH001** | Mouse monoclonal | Advanced Glycation End products (AGEs) |  | 1:500 | | Cosmo Bio Co. | |  |
